# Demographic determinants and effect of pre-operative angiotensin converting enzyme inhibitors and angiotensin receptor blockers on the occurrence of atrial fibrillation after CABG surgery

**DOI:** 10.1186/1471-2261-10-7

**Published:** 2010-02-08

**Authors:** Nasir Shariff, Steven Zelenkofske, Sherrine Eid, Michael J Weiss, Muneeruddin Q Mohammed

**Affiliations:** 1 Department of Medicine, Lehigh Valley Hospital, Allentown, Pennsylvania, USA; 2 Department of Cardiology, Lehigh Valley Heart Specialists, Lehigh Valley Hospital, Allentown, Pennsylvania, USA; 3 Department of Health Studies, Biostatistics, Lehigh Valley Hospital, Allentown, Pennsylvania, USA

## Abstract

**Background:**

Atrial fibrillation (AF) occurs in about 27% to 40% of post cardiac surgery patients. AF following coronary artery bypass graft surgery (CABG) is associated with a two-fold increase in morbidity and mortality. Various demographic risk factors and medications have been studied to predict the occurrence of this arrhythmia. The role of angiotensin related medications on the occurrence of AF in CABG patients is not determined.

**Methods:**

Retrospective clinical and statistical analysis was made of all the patients who had undergone CABG surgery at Lehigh Valley Hospital during the years 2005 and 2006. Patients with chronic AF and those undergoing valvular surgery with CABG were excluded. Statistic analysis included chi-square test for categorical and student t-test for continuous variables.

**Results:**

757 patients (560 males and 197 females) were studied. AF occurred in 19% of the patients. Age (70.5 vs. 65.1, p < 0.005. OR per year of age: 1.02, 95%CI: 1.018-1.023) and presence of hypertension (OR: 1.92, 95%CI: 1.086-3.140, p = 0.025) were significantly associated with occurrence of AF. Neither ARBs (OR: 0.78, 95%CI: 0.431-1.410, p = 0.41) nor ACE inhibitors (OR: 1.01, 95%CI: 0.753-1.608, p = 0.63) reduced the occurrence of post operative AF. Patients with post operative AF had a significantly longer hospital stay (9.5 +/- 5.4 days vs. 6.9 +/- 4.3 days, p = 0.001).

**Conclusions:**

Advanced age and presence of hypertension were independent predictors of post-CABG AF. Patients with post operative AF had significantly longer hospital stay. Neither ARBs nor ACE inhibitors were associated with reduction of post-surgical AF. Further studies are needed to better delineate the role of angiotensin related medications on reduction of post-surgical AF.

## Background

Atrial fibrillation (AF) occurs in about 27% to 40% of post cardiac surgery patients [1]. The presence of this arrhythmia following coronary artery bypass graft surgery (CABG) is associated with a two-fold increase in cardiovascular morbidity and mortality [2]. Post operative AF is associated with a higher occurrence of heart failure and cerebral ischemic accidents, both resulting in longer hospital stay, and consequently in higher hospital costs [3-6]. The etiology of postoperative AF is not well defined, although recent studies suggest a multi-factorial mechanism, which includes oxidative stress, inflammation, atrial fibrosis, excessive production of catecholamines, changes in autonomic tone and in the expression of connexins [7-11]. Multiple investigations have been performed to identify the demographic risk factors, association of medications and the predictors of post operative AF, but there is no conclusive information [12]. Epidemiological studies in non-surgical patients have shown that the use of angiotensin-converting enzyme inhibitors (ACEI) and angiotensin receptor blockers (ARBs) have an overall effect of 18% risk reduction in new-onset AF across the trials, and 43% risk reduction in patients with heart failure [13]. The present study was aimed at identifying the preoperative demographic predictors and the effects of ACEI and ARBs on the occurrence of AF in patients who underwent CABG surgery.

## Methods

A retrospective evaluation of patients who have undergone CABG surgery at Lehigh Valley Hospital was done. Patients were identified by ICD-9 surgical code for coronary artery bypass grafting. Data including baseline characteristics, past medical history, medicine use, and hospital course were extracted from an electronic medical record database. Each chart was reviewed and data entered by the investigators NS and MQM. Postoperative AF was defined as an entry into the case report form or by detection on the postoperative electrocardiogram. No distinction was made whether the arrhythmia was associated with symptoms or not. Details of medications received in the pre-operative period were noted. Included cases were divided into three groups: those on ACEI, those on ARBs and those on neither of these medications. Consecutive patients undergoing coronary arterial bypass surgery between January 2005 and December 2006 were included in the study. Patients younger than 18 years, those who were undergoing valvular surgery in addition to the CABG and patients with known AF at the time of going for the surgery were excluded from the study. Prior medical illness including hypertension and diabetes mellitus were studied as possible contributing causes of post surgical AF. Statistical analysis was conducted using SPSS 15.0 software. Group comparisons were performed using chi-square, t-test, ANOVA and non-parametric Kruskal-Wallace tests where appropriate. For those measures showing significant differences, appropriate odds ratios and 95% confidence intervals were calculated to provide ease of interpretation. The institutional review board at Lehigh Valley Hospital granted ethical approval for this study.

## Results

757 patients fulfilled the criteria and were included in the study. All the patients had on-pump CABG surgery. There were 560 males and 197 females. The mean age of the patients was 66.1 years with SD of 10.9 years. The average left ventricular ejection fraction (LVEF) was 51.2%. The duration of hospital stay ranged from 1 day to 45 days (mean 7.5 days with SD of 4.5 days). 634 (83%) patients were known to have hypertension, 606 (80%) were on beta-blockers, 260 (27%) on ACEI, and 94 (12%) were on ARBs. There were 262 (35%) patients with diabetes mellitus. 476 (63%) patients had history of smoking. 149 (20%) had COPD at the time of the surgery. Post operative AF occurred in 144 (19%) of the patients.

### Comparison of patients with and without AF following the CABG

Patients with AF following the CABG were noted to be older than those without the event, which was statistically significant (70.5 +/- 8.1 years vs. 65.1 +/- 11.2 years, p < 0.001). The odd ratio per year of age of developing AF after CABG was 1.02 with 95%CI of 1.018-1.023. Patients with AF had a significantly longer hospital stay (9.5 +/- 5.4 days vs. 6.9 +/- 4.3 days, p = 0.001). The occurrence of AF was not different in males as compared to females. There was no association of the arrhythmia with either smoking or history of COPD (Table 1). There was a significantly higher incidence of AF in hypertensive patients (OR: 1.92, 95%CI: 1.086-3.140, p = 0.025), but it was noted that on matching for age, this effect was lost. On subgroup analysis of hypertensive patients there was no association of occurrence of AF with the variables studied except as mentioned for age. Patients with diabetes mellitus had a higher incidence, while patients on ARBs had a lower incidence of AF, though neither were statistically significant. ACEI and beta-blockers had no effect on post-surgical AF, so was the case with Aspirin and Clopidogrel (Table 1).

**Table 1:** Comparison of discrete variables between patients who developed AF following CABG with those who did not have the arrhythmia following the surgery (Chi-squares test)

Particulars (patients in the group)	AF positive (n = 144)	AF negative (n = 613)	Odds Ratio	P value
Males (558)	107	456	0.97	0.89

Smoker (476)	86	390	0.87	0.45

COPD (149)	25	124	0.84	0.46

Hypertension (634)	129	505	1.92	0.017

Diabetes (262)	56	206	1.27	0.21

Aspirin (693)	133	560	1.16	0.66

Clopidogrel (261)	56	205	1.27	0.21

Beta-blockers (606)	118	488	1.17	0.51

ACEI(260)	52	208	1.01	0.75

ARB (94)	15	79	0.78	0.41

Statins (557)	107	450	1.28	0.71

Sternal infections (1)	1	0	-	0.38

Prolonged ventilation (27)	10	17	2.64	0.01

### Subgroup analysis of diabetic patients

On subgroup analysis of diabetic patients (Table 2), there was no association of AF with gender, smoking, or COPD. Again there was a noted higher incidence in hypertensive patients (p = 0.04). There was noted reduction in the occurrence of AF in patients who were on beta-blockers (p = 0.78) and ARBs (p = 0.45). It was also noted that there were fewer events patients on aspirin (p = 0.13). Patients with AF were noted to be older (71.2 +/- 8.6 years vs. 66.3 +/- 10.8 years, p = 0.002) and had a longer hospital stay (10.1 +/- 4.3 days vs. 7.7 +/- 5.3 days, p = 0.002).

**Table 2:** Comparison amongst diabetic patients of various factors that could contribute to post operative AF (n = 262).

Particulars (patients in the group)	AF positive (n = 56)	AF negative (n = 206)	Odds Ratio	P value
Males (184)	38	146	1.15	0.66

Smokers (147)	29	118	0.80	0.46

COPD (43)	5	38	0.43	0.09

Hypertension (240)	55	185	6.24	0.04

Aspirin (240)	49	191	0.47	0.13

Clopidogrel (90)	19	71	0.96	0.90

ARB (41)	7	34	0.71	0.45

ACEI (112)	26	86	1.19	0.57

Beta-blockers (212)	45	167	0.90	0.78

Statins (198)	44	154	1.57	0.56

### Subgroup analysis of patients who are older than 65 years with hypertension and diabetes

On additional subgroup analysis of these patients; no characteristic was identified with an increased occurrence of AF although there was a numerically lower propensity for AF in those patients receiving ARBs, clopidogrel, aspirin, beta-blockers and smokers although none of these was statistically significant (Table 3).

**Table 3:** Comparison of various factors amongst patients older than 65 years, positive history of hypertension, and diabetes for occurrence of post-operative AF (n = 157).

Particulars	AF positive (n = 56)	AF negative (n = 206)	Odds Ratio	P value
Males (108)	29	79	0.98	0.97

Smokers (85)	20	65	0.70	0.32

Aspirin (143)	37	106	0.63	0.42

Clopidogrel (57)	13	44	0.72	0.40

ARB (32)	5	27	0.44	0.11

ACEI (71)	19	52	1.01	0.99

Beta-blockers (131)	34	97	0.79	0.61

Statins (120)	33	87	1.70	0.50

### Comparison of patients on ACEI or ARBs with those who were not on these medications

260 patients were on ACEI, 94 were on ARBs and 403 patients were on neither of these medications. AF occurred in 20% of patients on neither ACEI nor ARBs. 20% of patients on ACEI and 16% of patients on ARBs had post-operative AF. Although numerically there was less occurrence of AF in patients on ARBs, this was not statistical significant (OR: 0.78, 95%CI: 0.431-1.410, p = 0.41). We did note that patients on ARBs were significantly older than patients who were not on ARBs (68.2 +/- 9.2 yrs vs. 65.8 +/- 11.1 yrs. p = 0.05). Further evaluation with matching for age showed no significant effect of ARBs on occurrence of AF. Patients who were noted to benefit the most were those between 80 and 89 years of age (21% vs 30%, p = 0.15). Of the 28 patients in this age group who were on ARBs, 6 had post operative AF as against 59 of 199 patients who were not on this medication.

## Discussion

AF is a common cardiac arrhythmia following cardiothoracic surgery. AF occurs in about 27% to 40% of post cardiac surgery patients [1]. AF most frequently occurs in the first 2 to 3 days after cardiothoracic surgery [14]. The presence of this arrhythmia following coronary artery bypass graft surgery is associated with a two-fold increase in cardiovascular morbidity and mortality [2]. There have been various studies suggesting the positive effects of ACEI and ARBs on the occurrence of this complication, though it is still controversial [15,16].

In our retrospective study, we looked at patients who underwent CABG without valvular surgery, on the contributing factors and effects of various cardiac medications on the occurrence of post surgical AF. In our analysis AF occurred in 19% of patients. The reduced incidence of AF amongst our patients as compared to other quoted studies [1,15,16] is possibly attributed to the exclusion of patients who were also undergoing valvular surgery, which is known to increase the incidence of the AF. Increasing age was noted to be associated with higher occurrence of AF, whereas gender, diabetes, and left ventricular ejection fraction were not associated with AF. Patients with AF had a significantly longer hospital stay (9.5 +/- 5.4 days vs. 6.9 +/- 4.3 days, p = 0.001). The noted influence of age as the positive predictor of occurrence of AF has been noted previously [17]. We did see a higher occurrence of AF among patients with hypertension, but it was noted that on matching to age, this effect was lost. This finding reaffirming the role of age was unique to our study.

Use of ACEI and ARBs as a medication to reduce AF following CABG has not been fully studied to date. ACEI and ARBs are routinely used perioperatively and if they were to provide protection against AF, this would provide additional rationale to using these agents in all patients undergoing CABG. The Renin-Angiotensin system has been shown to play a key role in vascular inflammation, cardiac remodelling, fibrosis and apoptosis, and autonomic control. Agents that block the renin-angiotensin system have been reported not only to prevent left atrial dilation and atrial fibrosis but also to slow conduction velocity in the heart, explaining the reduction of AF in post CABG patients [18-20]. At the same time there are reports of increase in AF with these agents [15]. An explanation for the increased occurrence of AF in post-operative patients on ACEI and ARBs is thought to be attributed to the higher prevalence of hypertension and left ventricular hypertrophy in these patients [15].

In our study we compared the effects of ACEI and ARBs as separate groups on the occurrence of AF; this has not been done previously. Interestingly we noted that though not statistically significant, patients on ARBs had fewer occurrences of AF post operatively unlike patients who were on ACEI. Similar differences have been noted between the two medications in relation to reduction of cerebro-vascular accidents [21]. The likely explanation for this finding is not known, though there are a few hypothetical explanations for its occurrence. ACEI antagonize the effects of AT-1 and AT-2 receptors while ARBs block the AT-1 and stimulate AT-2 receptors. Stimulation of AT-2 receptors counteracts some effects of AT-1 receptors and may have antiproliferative and cardioprotective action. In addition the ACEI do not affect angiotensin II production via non-ACE pathways, whereas ARBs antagonize all effects consequent upon AT-1 receptor activation. Chymase activity (non-ACE pathway) is present in the human heart tissue extract and is higher in the left atrium than in other chambers [22]. In a recent study, treatment with Valsartan was not associated with a reduction in the incidence of recurrent AF [23]. This study enrolled patients who were in sinus rhythm but had previous documented episodes of AF and they looked at the incidence of reoccurrence of the arrhythmia. Half of the studied patients were on other antiarrhythmic medications like amiodarone, sotalol and class I antiarrhythmic agents, which was quite in contrast to our study population. Though there was only a marginal reduction of occurrence of AF in the treated population in that study, we could speculate that there could have been a bigger benefit in medication naive patients. It is also well established that the etiology of AF following CABG is very different from non-surgical patients who were studied in the GISSI-AF trial. In our study we note that patients on ARBs were significantly older than patients who were not on ARBs. Since we had seen that age was significantly associated with occurrence of AF; further evaluation with matching the age was done. On matching for age - there was no significant reduction of incidence of occurrence of AF in any particular age group though patients between the age of 80 and 89 years noted to be most benefited with ARBs.

Our study had some limitations; firstly, we were not able to assess left atrial size and left ventricular hypertrophy which are known risk factors of post operative AF, and would recommend that this data be included in further studies. Secondly, as this was a cohort study, investigators did not dictate the use of ACEI or ARB or its dosages, nor had any control on the duration of medications. This lack of randomisation in observational studies could introduce both confounding factors and biases. We addressed most of the confirmed risk factors by matching patients in our statistical analysis.

## Conclusion

Advanced age and presence of hypertension were independent predictors of post-CABG AF. Patients with post operative AF had significantly longer hospital stay. Neither ARBs nor ACE inhibitors were associated with reduction of post-surgical AF. Further studies are needed to better delineate the role of angiotensin related medications on reduction of post-surgical AF.

## Competing interests

The authors declare that they have no competing interests.

## Authors' contributions

NS was involved with design of the study, collecting the data, coordination and drafting the manuscript. SZ was involved in drafting the manuscript. MQM was involved in collection of the data and drafting the manuscript. SE participated in the design of the study and performed the statistical analysis. MJW was involved in performing the statistical analysis and drafting the manuscript. All authors read and approved the final manuscript.

## Pre-publication history

The pre-publication history for this paper can be accessed here:

http://www.biomedcentral.com/1471-2261/10/7/prepub
